# Chronic Lymphocytic Leukemia: A Rare Cause of Pathological Fracture of the Femur

**DOI:** 10.1177/2324709617735135

**Published:** 2017-10-03

**Authors:** Parita Soni, Nidhi Aggarwal, Anand Rai, Vivek Kumar, Kamholz Stephan, Yoon Taek, Kupfer Yizhak

**Affiliations:** 1 Maimonides Medical Center, Brooklyn, NY, USA

**Keywords:** chronic lymphocytic leukemia, pathological fracture, hypercalcemia, Richter’s transformation, multiple myeloma

## Abstract

The incidence rate of chronic lymphocytic leukemia (CLL) in the United States is approximately 0.005%; men are at slightly higher risk than women. Bony involvement or pathological fracture rarely occurs in CLL, and it may be the initial presentation. An 85-year-old woman presented with acute respiratory failure secondary to pneumonia. Symptomatology included dyspnea. She was found to have pathological fracture of the femur caused by CLL. The diagnosis of CLL had been made 6 years previously, but the patient had refused therapy. On admission, the patient required endotracheal intubation, mechanical ventilation, and admission to the medical intensive care unit. Endotracheal intubation extubation was successful after 48 hours. The patient then complained of severe left knee pain. Bone radiograph and femoral computed tomography scan revealed acute pathological fracture of the left distal femur. There was no history of trauma. The fracture was stabilized with extension lock splint. Pathological fracture in patients with CLL is associated with hypercalcemia, Richter’s transformation, or multiple myeloma. This patient exemplifies the fact that pathological fracture can be caused by CLL in the absence of hypercalcemia, Richter’s transformation, or multiple myeloma and can be the initial presentation of CLL.

## Introduction

Chronic lymphocytic leukemia (CLL) is a lymphoproliferative disorder mainly affecting older adults. The average age at diagnosis is 71 years.^1^ CLL accounts for one quarter of the new cases of leukemia and affects every 1 in 200 persons (about 0.005%) in the United States.^2^ The presentation varies from completely asymptomatic to the eventual development of anemia, thrombocytopenia, and infections due to involvement of lymph nodes, spleen, and liver. Bony involvement in leukemia is most commonly associated with acute lymphoblastic or acute myeloblastic leukemia.^3^ CLL leading to pathological fracture is a rare phenomenon.^4^ We present a patient with stage III CLL causing a pathological fracture of the distal femur.

## Case Presentation

An 85-year-old Caucasian woman presented to the emergency department with shortness of breath. She was diagnosed with CLL 6 years ago, and she also had atrial fibrillation, chronic heart failure, and hypertension. She had refused therapy at the time of initial diagnosis. On presentation, temperature was 101.3°F, heart rate 130/min, respiratory rate 32/min, blood pressure 156/67 mm Hg, and SpaO_2_ 80% on ambient air, which improved minimally after the institution of BiPAP. In the emergency department, the patient was intubated because of acute respiratory failure and sepsis secondary to pneumonia. She was admitted to the medical intensive care unit and was successfully extubated after 2 days of treatment.

**Figure 1.**
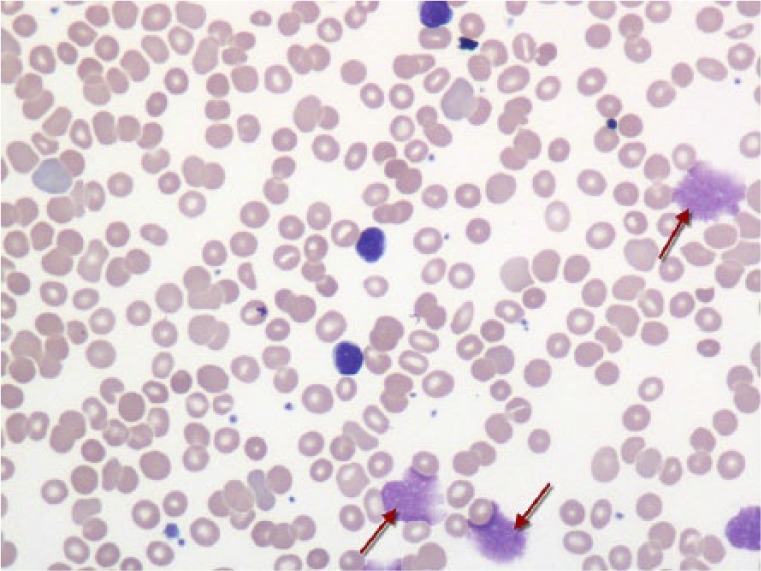
Peripheral smear (60×) showing small lymphocytes and smudge cells (arrows).

Laboratory work data included white blood cells count 107 000/µL, hemoglobin 10.4 g/dL, and platelets 149 000/µL. Peripheral smear revealed 2+ smudge cells and more than 7 atypical lymphocytes per oil immersion field (Figure 1). Electrolyte panel was within normal limits. Bone marrow examination revealed no signs of Richter’s transformation or multiple myeloma. The patient refused treatment for CLL in the past but intravenous immunoglobulin therapy was instituted because of the risk of serious infectious complications.

**Figure 2.**
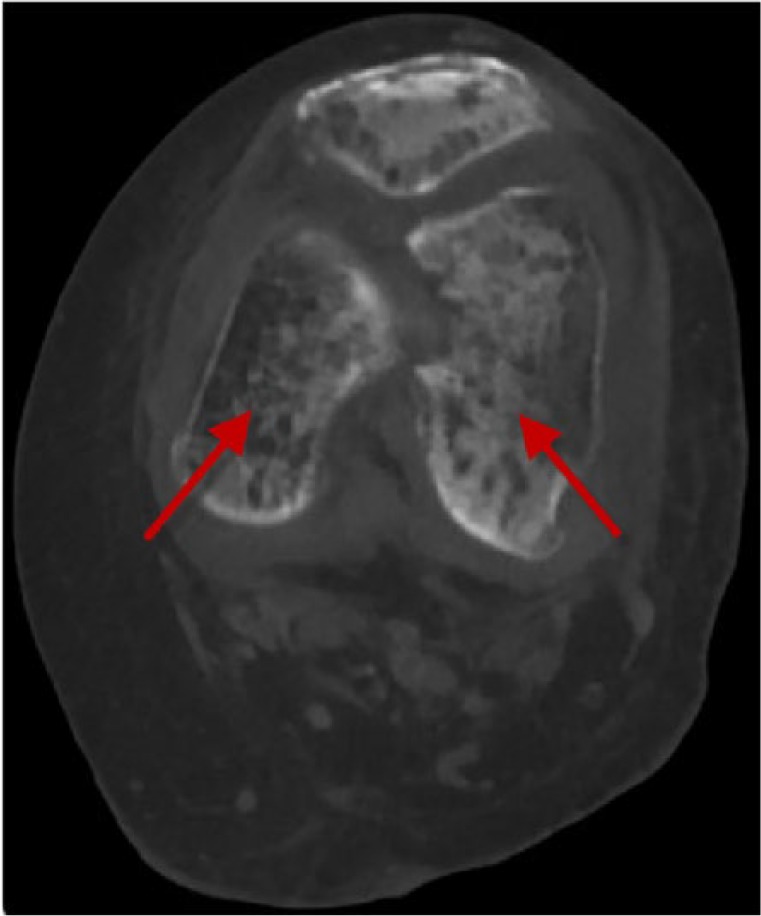
Enhanced computed tomography of the left knee showing acute pathological fracture of the left distal femur (arrows).

On hospital day 3, she began complaining of severe left knee pain. She denied any trauma or fall. The left knee was warm and extremely tender to touch. Arthrocentesis excluded the possibility of septic arthritis. Bone radiograph demonstrated an acute pathological fracture of the left distal femur. This finding was confirmed by computed tomography of the left knee (Figure 2).

Although open reduction internal fixation is the treatment of choice, it was not recommended because of her limited prognosis attributable to the nature of the fracture, severe osteopenia, and other comorbidities. The patient was treated conservatively for fracture stabilization with extension lock splint, and her pain was controlled with the pain medications. She was then discharged home in a stable condition.

## Discussion

CLL is a monoclonal disorder characterized by progressive accumulation of functionally incompetent lymphocytes. Usually the early stage of CLL requires no treatment unless the patient is symptomatic or there is a rapid disease progression.^1^ Patients with CLL are at high risk of developing common infections (upper and lower respiratory infections), immune system problems, complex/unusual infections, and are at increased risk of developing other cancers.^5-7^ Patients with untreated CLL have increased risk of developing spine and pelvic fractures due to altered bone metabolism, resorption, and demineralization.^1,8^ However, CLL causing pathological fracture of the long bones is rare.^4^

Bony involvement in patients with leukemia is often associated with acute lymphoblastic or acute myeloblastic leukemia.^3^ Prior reports of pathological fracture due to CLL involved the spine and vertebral compression fractures and were found to be associated with hypercalcemia.^9-12^ Langenberg et al reported a patient who was hypercalcemic and did not show any signs of Richter’s transformation or multiple myeloma on the bone marrow sample but still developed pathological fracture of the distal femur due to CLL.^13^ Almost all of the previously reported cases of pathological fracture associated with CLL occurred in the presence of hypercalcemia and were associated with Richter’s transformation or multiple myeloma.^9-16^

Review of literature suggests that the exact pathophysiology of the pathological fracture in CLL is not known but may be secondary to locally released osteoclast stimulating factors.^14^ It may be appropriate to include pathological long bone fracture in the diagnostic assessment of CLL patients complaining of bone/joint pain.
